# Adjuvant Radiotherapy and Survival in Parotid Gland Adenoid Cystic Carcinoma: A National Cancer Database Analysis

**DOI:** 10.1002/ohn.70264

**Published:** 2026-04-27

**Authors:** Evan Kominsky, Marium Ansari, Michael Moore, Michael Sim, Avinash Mantravadi, Jessica Yesensky, Janice L. Farlow

**Affiliations:** ^1^ Department of Otolaryngology Indiana University School of Medicine Indianapolis Indiana USA; ^2^ Indiana University School of Medicine Indianapolis Indiana USA

**Keywords:** adenoid cystic carcinoma, adjuvant radiation, overall survival, parotid, radiotherapy

## Abstract

**Objective:**

To determine the association of positive margins and adjuvant radiotherapy on overall survival in parotid gland adenoid cystic carcinoma.

**Study Design:**

Retrospective cohort analysis.

**Setting:**

National Cancer Database from 2004 to 2021.

**Methods:**

The National Cancer Database was queried for patients diagnosed with adenoid cystic carcinoma of the parotid who underwent upfront definitive surgical excision. Kaplan‐Meier and Cox proportional hazard modeling were used for data analysis.

**Results:**

Of 1360 included subjects, the mean age was 56.1 years (range 18‐90), 61.6% were female, and 80.4% were white. Negative margins were reported in 52.0%, with microscopic positive margins present in 29.4% of patients. Total parotidectomy was performed in 52.4%, with facial nerve sacrifice in 36.0%. Adjuvant radiotherapy was given to 78.2% and chemotherapy to 8.7%. Median follow‐up was 83.4 months; 10‐year overall survival was 63.1%. Patients with positive margins, older age, male sex, and advancing stage were associated with worse overall survival. Adjuvant radiotherapy did not improve overall survival in the entire cohort (*P* = .8) or in early‐stage disease (pT1N0‐pT2N0) (*P* = .15) or microscopically positive margin subgroups (*P* = .8).

**Conclusion:**

Surgically resected adenoid cystic carcinoma of the parotid gland frequently exhibits positive margins, with adjuvant radiotherapy given in the majority of cases. With the inherent limitations of retrospective analyses of the National Cancer Database, radiotherapy had no significant difference in 10‐year overall survival.

Adenoid cystic carcinoma (ACC) of the parotid gland is a rare malignancy with poor long‐term survival. While it often exhibits a slow clinical course, its poor prognosis is reflected by frequent perineural invasion, and high rates of local recurrence and microsatellites of disease.^1^, ^2^ Despite advances in diagnosis and identification of molecular therapeutic targets, long‐term survival remains poor, and optimal management strategies, particularly regarding adjuvant therapy, are still debated. For surgically resectable tumors, parotidectomy is the mainstay of treatment. Current National Cancer Care Network (NCCN) Guidelines recommend adjuvant radiotherapy (RT) for surgically resected disease even in the absence of the above‐mentioned pathologic features, although it is unclear of the associated survival benefit for adjuvant RT.^3^, ^4^, ^5^

Due to the rarity of ACC of the parotid gland and lack of consensus about the benefit of postoperative RT on overall survival (OS), we performed a retrospective cohort analysis of the National Cancer Database (NCDB) among patients with primary parotid gland ACC who underwent initial surgical resection.

Our primary objective was to determine the role of adjuvant RT on 10‐year OS in patients who underwent primary surgical resection of ACC of the parotid gland. Our secondary objective was to determine additional factors that influence OS in this cohort.

## Methods

The current study was reviewed and determined to be exempt from Institutional Review Board (IRB) oversight. The STROBE reporting guidelines were followed.

### Study Design, Setting, and Patient Selection

A retrospective cohort analysis was performed using patients diagnosed with ACC included in the NCDB between 2004 and 2021. The NCDB is a hospital‐based oncology database with more than 1500 Commission on Cancer‐accredited facilities in the United States and is jointly administered by the American College of Surgeons and the American Cancer Society. The database provides information about patient demographics, treatments, and outcomes of about 70% of newly diagnosed malignancies annually.

**Figure 1:**
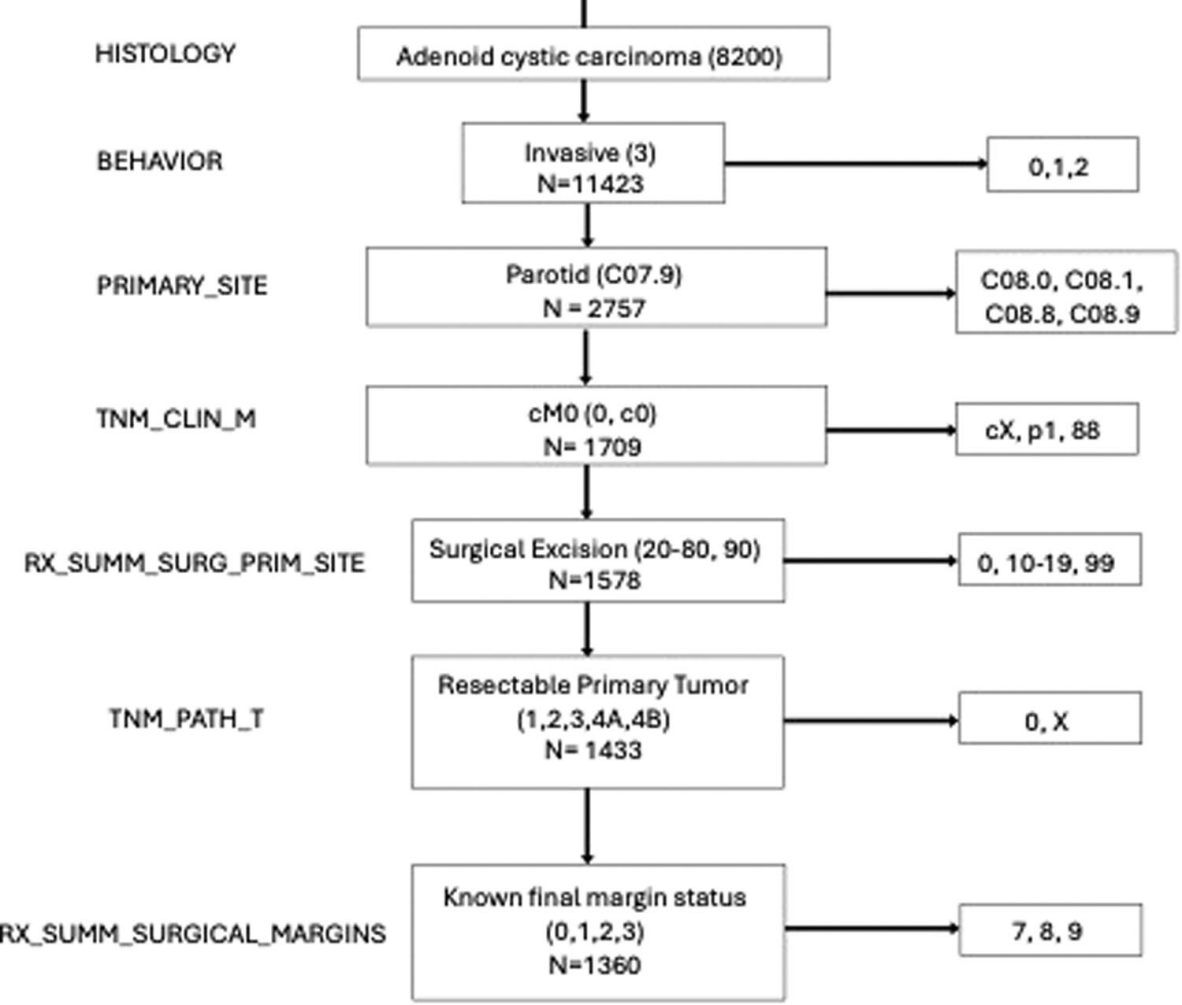
Inclusion‐exclusion flowchart. PUF, participant use file.

Inclusion criteria consisted of patients ≥ 18 years of age; diagnosis of primary ACC of the parotid gland corresponding to the International Classification of Diseases for Oncology 3rd Edition (ICD‐O‐3) code 8300; cT1‐4, any cN, and cM0. We excluded patients who were not treated with surgical intervention, had distant metastasis, and for whom the final surgical margin status was unknown due to incomplete or missing records (Figure 1).

Staging was reported per the American Joint Committee on Cancer 7th or 8th Edition depending on the date of diagnosis. The following patient characteristics were examined: sex, age, race, insurance status, type of treatment hospital (academic vs community), and Charlson‐Deyo comorbidity score.^6^, ^7^ Tumor characteristics examined included: size, presence of lymphovascular invasion, TNM category, and overall stage. Treatment characteristics examined included the following: days from diagnosis to treatment, extent of surgery, facial nerve sacrifice, surgical margin status, receipt of adjuvant RT, and receipt of adjuvant chemotherapy.

### Statistical Methods

OS was analyzed using the Kaplan‐Meier method, with differences between groups compared using the log‐rank test. Univariate and multivariate Cox proportional hazards regression models were used to identify predictors of OS, with hazard ratios (HRs) and 95% confidence intervals (CIs) reported. For all statistical tests, a *P*‐value of <.05 was considered significant. Analyses were performed using R version 4.4.1. Missing or incomplete data were reported where applicable in the descriptive statistics and were excluded in the case of regression modeling where necessary.

## Results

### Patient Characteristics

A total of 1360 patients who met study criteria were identified (Table 1). The cohort had a mean age of 56.1 years (SD 15.9) and was predominantly female (61.6%). Most patients were white (80.4%), and the majority had private (55.4%) insurance coverage. At the time of surgical resection, the mean tumor size was 28.2 mm (SD 18.4). Margin status was positive in 48% of cases. Of the positive margin group, 1.8% were reported to have macroscopically positive margins, 29.4% microscopically positive, and 16.8% with positive margins of unspecified nature. The distribution of pathologic T category was: T1 (27.1%), T2 (22.9%), T3 (19.5%), and T4A/B (26.5%). Of those patients who underwent a regional lymph node dissection (RLND) (76.1%), the pathologic nodal category revealed that most patients were noted to have N0 disease (77.3%); 16.4% of patients had unknown nodal status due to missing or incomplete data. Total parotidectomy was performed in 52.4% of cases, while superficial and radical parotidectomies were performed in 37.7% and 9.9% of cases, respectively. Neck dissection was conducted in 76.1% of patients. Facial nerve sacrifice was documented in 36%. Adjuvant RT was administered in 78.2% of cases, and chemotherapy in 8.7%.

**Table 1:** Patient Cohort Characteristics

	No adjuvant RT	Adjuvant RT	Overall
	(N = 297)	(N = 1063)	(N = 1360)
Age			
Mean (SD)	58.7 (17.0)	55.4 (15.6)	56.1 (15.9)
Median [Min, Max]	60.0 [21.0, 90.0]	56.0 [18.0, 90.0]	57.0 [18.0, 90.0]
Sex			
Female	182 (61.3%)	656 (61.7%)	838 (61.6%)
Male	115 (38.7%)	407 (38.3%)	522 (38.4%)
Race			
Black	34 (11.4%)	107 (10.1%)	141 (10.4%)
Other	26 (8.8%)	100 (9.4%)	126 (9.3%)
White	237 (79.8%)	856 (80.5%)	1093 (80.4%)
Insurance status			
Public	141 (47.5%)	395 (37.2%)	536 (39.4%)
Private	136 (45.8%)	618 (58.1%)	754 (55.4%)
Uninsured	15 (5.1%)	30 (2.8%)	45 (3.3%)
Unknown	5 (1.7%)	20 (1.9%)	25 (1.8%)
CD score			
0	258 (86.9%)	886 (83.3%)	1144 (84.1%)
1+	39 (13.1%)	177 (16.7%)	216 (15.9%)
Tumor size, mm			
Mean (SD)	26.8 (17.7)	28.6 (18.6)	28.2 (18.4)
Median [Min, Max]	22.0 [1.00, 160]	25.0 [2.00, 250]	25.0 [1.00, 250]
Missing	25 (8.4%)	82 (7.7%)	107 (7.9%)
Lymphovascular invasion			
No	147 (49.5%)	443 (41.7%)	590 (43.4%)
Yes	17 (5.7%)	109 (10.3%)	126 (9.3%)
Missing	133 (44.8%)	511 (48.1%)	644 (47.4%)
Margin status			
Negative	207 (69.7%)	500 (47.0%)	707 (52.0%)
Positive (macroscopic)	5 (1.7%)	20 (1.9%)	25 (1.8%)
Positive (microscopic)	57 (19.2%)	343 (32.3%)	400 (29.4%)
Positive (unspecified)	28 (9.4%)	200 (18.8%)	228 (16.8%)
Pathologic T category			
T1	106 (35.7%)	262 (24.6%)	368 (27.1%)
T2	73 (24.6%)	239 (22.5%)	312 (22.9%)
T3	50 (16.8%)	215 (20.2%)	265 (19.5%)
T4A/B	50 (16.8%)	311 (29.3%)	361 (26.5%)
Missing	18 (6.1%)	36 (3.4%)	54 (4.0%)
Pathologic N category^a^			
cN0	6 (2.0%)	9 (0.8%)	15 (1.1%)
pN0	201 (67.7%)	724 (68.1%)	925 (68.0%)
pN1	19 (6.4%)	107 (10.1%)	126 (9.3%)
pN2	13 (4.4%)	57 (5.4%)	70 (5.1%)
pN3	0 (0%)	1 (0.1%)	1 (0.1%)
pNX	39 (13.1%)	113 (10.6%)	152 (11.2%)
Missing	19 (6.4%)	52 (4.9%)	71 (5.2%)
Overall stage			
I	85 (28.6%)	213 (20.0%)	298 (21.9%)
II	60 (20.2%)	181 (17.0%)	241 (17.7%)
III	44 (14.8%)	218 (20.5%)	262 (19.3%)
IV	50 (16.8%)	318 (29.9%)	368 (27.1%)
Missing	58 (19.5%)	133 (12.5%)	191 (14.0%)
Type of parotidectomy			
Radical	19 (6.4%)	115 (10.8%)	134 (9.9%)
Superficial	135 (45.5%)	378 (35.6%)	513 (37.7%)
Total	143 (48.1%)	570 (53.6%)	713 (52.4%)
RLND performed			
No	81 (27.3%)	239 (22.5%)	320 (23.5%)
Yes	215 (72.4%)	820 (77.1%)	1035 (76.1%)
Missing	1 (0.3%)	4 (0.4%)	5 (0.4%)
Facial nerve sacrifice			
No	200 (67.3%)	671 (63.1%)	871 (64.0%)
Yes	97 (32.7%)	392 (36.9%)	489 (36.0%)
Chemotherapy			
No	289 (97.3%)	928 (87.3%)	1217 (89.5%)
Yes	4 (1.3%)	114 (10.7%)	118 (8.7%)
Missing	4 (1.3%)	21 (2.0%)	25 (1.8%)
Follow‐up, mo			
Mean (SD)	81.9 (48.0)	91.4 (48.0)	89.4 (48.1)
Median [Min, Max]	76.9 [0.330, 214]	85.9 [2.27, 227]	83.4 [0.330, 227]

Abbreviations: CD, Charlson‐Deyo; RLND, regional lymph node dissection; RT, radiotherapy; SD, standard deviation.

^a^ For patients who did not undergo a regional lymph node dissection and where clinically N0, there final nodal status was reported as cN0.

### Survival Outcomes

**Figure 2:**
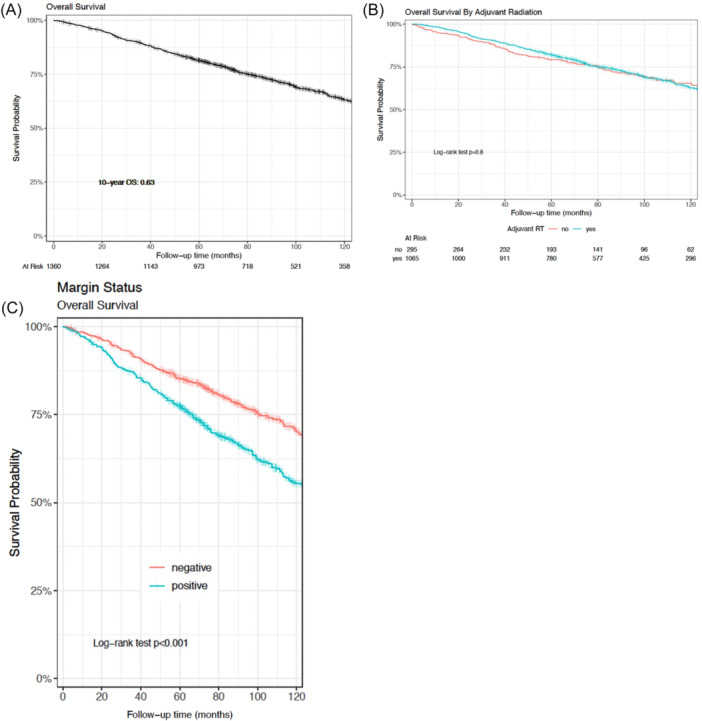
Kaplan‐Meier survival curves. Total cohort (A), stratified by adjuvant radiotherapy (B), and postoperative margin status (C). OS, overall survival; RT, radiotherapy.

The median follow‐up time was 83.4 months. The 10‐year OS was 63.1%. Kaplan‐Meier analysis revealed that margin status significantly impacted survival (log‐rank *P* < .001) (Figure 2). The receipt of adjuvant RT was not associated with OS (log‐rank *P* = .8).

In univariate Cox regression, the following factors were significantly associated with worse OS: increasing age (HR = 1.04, *P* < .001), male sex (HR = 1.39, *P* = .0007), Charlson‐Deyo score ≥ 1 (HR = 2.06, *P* < .001), positive surgical margins (HR = 1.66, *P* < .001), locally advanced disease (HR = 2.30, *P* < .001), higher overall pathologic stage (HR = 3.93, *P* < .001 for stage IV), total (HR = 1.52, *P* < .001) or radical (HR = 2.42, *P* < .001) parotidectomy, facial nerve sacrifice (HR = 1.61, *P* < .001), and chemotherapy (HR = 1.49, *P* = .01).

In the multivariable model (Table 2), pathologic stage was used as a stratification factor due to violation of the proportional hazard assumption. The following variables remained significantly predictive of worse OS: age (HR = 1.04, *P* < .001), male sex (HR = 1.26, *P* = .026), Charlson‐Deyo score ≥ 1 (HR = 1.67, *P* < .001), and positive margins (HR = 1.43, *P* = .001). Surgery extent and chemotherapy were not statistically significant in the multivariate model.

**Table 2:** Cox Proportional Hazard Multivariate Model^a^

Characteristics	HR	95% CI	*P*‐value
Age	1.04	1.03, 1.05	<.001
Sex			
Female	‐	‐	
Male	1.26	1.03, 1.55	.026
Charlson‐Deyo score			
0	‐	‐	
1+	1.67	1.31, 2.13	<.001
Margin status			
Negative	‐	‐	
Positive	1.43	1.15, 1.77	.001
Surgery extent			
Radical	1.35	0.92, 1.99	.13
Superficial	‐	‐	
Total	1.26	0.99, 1.61	.061
Facial nerve sacrifice			
No	‐	‐	
Yes	1.04	0.82, 1.33	.7
Chemotherapy			
No	‐	‐	
Yes	1.27	0.90, 1.79	.2

Abbreviations: CI, confidence interval; HR, hazard ratio.

^a^ Pathological stage was used as a stratification variable due to violation of the proportional hazard assumption.

### Subgroup Analyses

**Figure 3:**
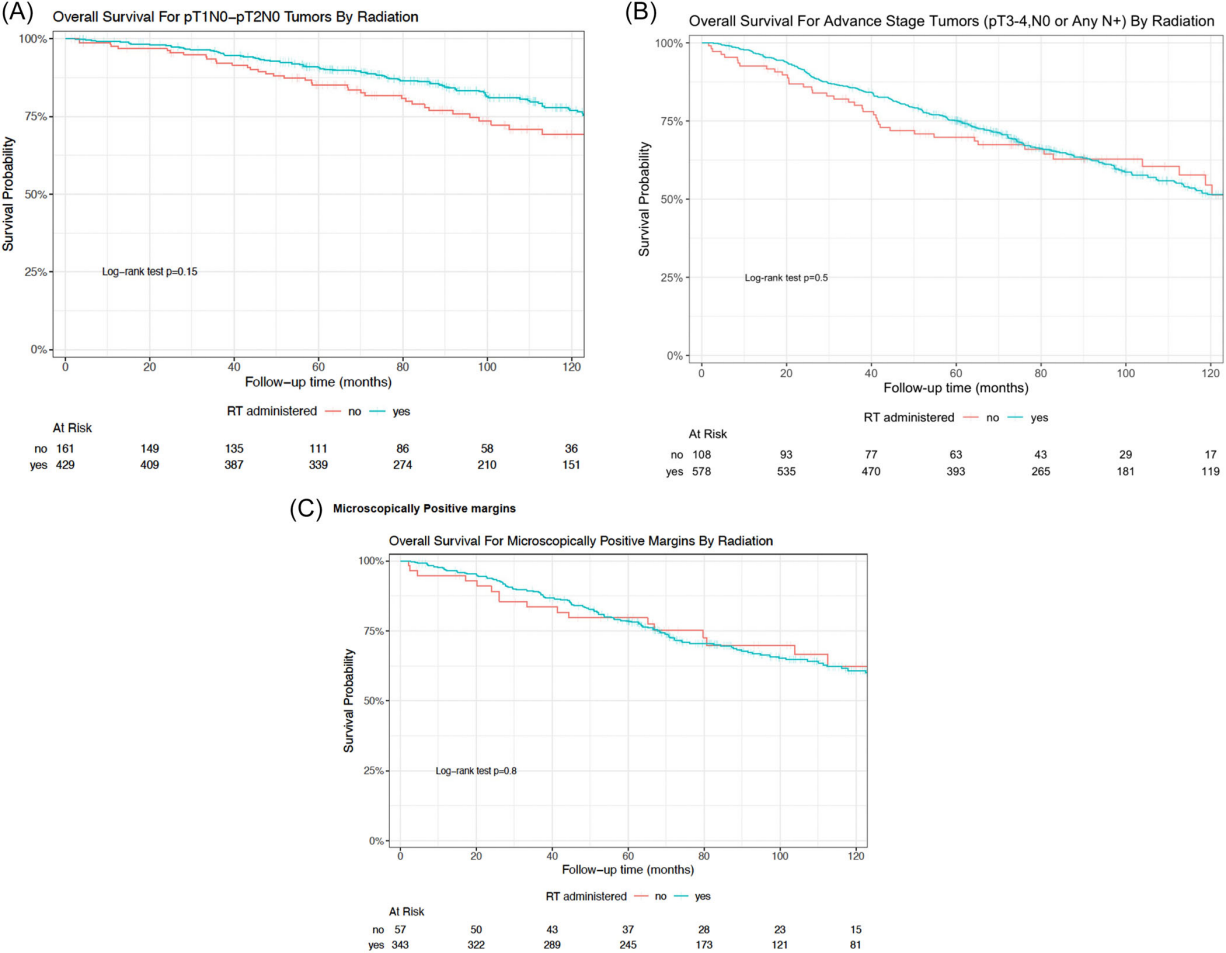
Kaplan‐Meier survival curves. Subgroup analysis of early stage (T1‐T2N0) (A), advanced stage (T3‐4 or any N+) (B), and microscopically positive margins stratified by adjuvant radiotherapy (RT) (C).

Among 590 patients with early‐stage disease (pT1‐pT2N0), 72.7% received adjuvant RT; this was not associated with OS (log‐rank *P* = .15; HR = 0.76, 95% CI: 0.52‐1.11) (Figure 3A). The advanced‐stage disease subgroup (pT3‐pT4N0 or N+), which contained 686 patients, did not show a survival difference for adjuvant radiation (log‐rank *P* = .5) (Figure 3B). Similarly, in patients with microscopically positive margins, there was no observed association between survival and adjuvant RT (log‐rank *P* = .8) (Figure 3C).

## Discussion

In this cohort of 1360 patients with primary ACC of the parotid gland treated with surgical resection, we found that 78.2% received adjuvant RT without any significant difference in 10‐year OS (log‐rank *P* = .8). There have been no prior studies whose outcomes focus exclusively on ACC of the parotid gland, which makes direct comparison of outcomes difficult. However, our finding is consistent with several prior national database studies, which included ACC of all head and neck subsites that have shown no benefit of adjuvant RT on OS or disease‐specific survival.^8^, ^9^, ^10^ The effect of adjuvant RT on locoregional control (LRC) is not well studied, and to our knowledge, there is no publicly available study that directly compares this. Neither data about LRC nor disease‐free survival are available in the NCDB; only OS can be examined in this data set, which remains a limitation of our analysis. Additionally, the possibility of a type II error must be considered. This study includes one of the largest cohorts of surgically treated parotid gland ACC reported to date, with long‐term follow‐up and a substantial number of survival events in both adjuvant and non‐adjuvant groups. The observed HRs and corresponding CIs suggest that a large survival benefit from adjuvant RT is unlikely, though a more modest effect size cannot be definitively excluded. Given the indolent natural history and late recurrence pattern of ACC, even larger cohorts or longer follow‐up may be required to detect such small differences in OS.

As noted, previous large database studies of head and neck ACC have analyzed all subsites together. We chose to focus exclusively on parotid ACC for several reasons. We anticipated that a more homogenous disease population would provide for more robust outcomes from our analysis that would be more clinically applicable. For parotid neoplasms, the extent of parotidectomy, decision‐making surrounding facial nerve sacrifice, and adjuvant treatment consideration are frequent clinical and surgical challenges that distinguish the parotid from other primary sites. While previous studies have reported on ACC outcomes using the NCDB data, our study includes an additional 9 years of new cases and follow‐up, including cases from 2004 to 2021.

Our subgroup analyses of early‐stage disease (pT1‐T2N0) and microscopically positive margins found that adjuvant RT was not associated with OS (*P* = .15 and *P* = .8, respectively). These findings contrast with Lee et al, whose NCDB analysis did report a 5‐year OS benefit in the early‐stage disease subgroup.^5^ However, Lee et al include all head and neck subsites and used data from 2004 to 2012. We surmised that the effect that was observed in their study may have been lost with the increased follow‐up time given the tendency for recurrences to present in a delayed and indolent fashion.

Our study provides a relatively large sample size for a rare tumor type as well as a long follow‐up time that is typically only available in large multicenter databases. However, limitations of this analysis include its retrospective design that cannot capture the nuances of adjuvant treatment recommendations and adherence, as well as the inherent constraints of NCDB data including a lack of information on LRC and disease‐specific survival. Data in the NCDB are also subject to coding issues, missingness, and a lack of data on cause of death. An additional source of bias in survival analysis is immortal time bias. In our study, this would include the time between surgical intervention and the start of adjuvant radiation for those patients in the adjuvant RT group. Given the long follow‐up duration of our study and ACC's indolent growth pattern, we would not expect the immortal time in the non‐RT group to be a significant contributor to a change in OS. ACC is characterized by indolent but relentless progression and late recurrences, and our median follow‐up of nearly seven years may still be insufficient to capture the full impact of adjuvant therapies. Additionally, the decision to pursue adjuvant radiation is multifaceted and may have implications on LRC and patient‐specific factors not addressed in the current study. As always, shared decision‐making and multidisciplinary tumor board discussion should be the drivers for complex patient care decisions.

## Conclusions

While our findings do not support an OS benefit for adjuvant RT in parotid ACC, they reaffirm the prognostic significance of age, comorbidity, sex, and margin status on overall patient survival. Given conflicting evidence, prospective multicenter studies are needed to clarify the optimal use of adjuvant RT in this rare malignancy.

## Author Contributions

**Evan Kominsky**: Conceptualization, methodology, formal analysis, writing—original draft, visualization. **Marium Ansari**: Writing—original draft, visualization. **Michael Moore**: Investigation, formal analysis, writing—original draft, writing—review and editing. **Michael Sim**: Formal analysis, writing—original draft, writing—review and editing. **Avinash Mantravadi**: Formal analysis, writing—original draft, writing—review and editing. **Jessica Yesensky**: Formal analysis, writing—original draft, writing—review and editing. **Janice L. Farlow**: Conceptualization, methodology, formal analysis, supervision.

## Disclosures

### Competing interests

The authors declare no conflicts of interest.

### Funding source

None.
